# Chirality-dependent flutter of *Typha* blades in wind

**DOI:** 10.1038/srep28907

**Published:** 2016-07-19

**Authors:** Zi-Long Zhao, Zong-Yuan Liu, Xi-Qiao Feng

**Affiliations:** 1 AML, Institute of Biomechanics and Medical Engineering, Department of Engineering Mechanics, Tsinghua University, Beijing 100084, China; 2 Center for Nano and Micro Mechanics, Tsinghua University, Beijing 100084, China

## Abstract

Cattail or *Typha*, an emergent aquatic macrophyte widely distributed in lakes and other shallow water areas, has slender blades with a chiral morphology. The wind-resilient *Typha* blades can produce distinct hydraulic resistance for ecosystem functions. However, their stem may rupture and dislodge in excessive wind drag. In this paper, we combine fluid dynamics simulations and experimental measurements to investigate the aeroelastic behavior of *Typha* blades in wind. It is found that the chirality-dependent flutter, including wind-induced rotation and torsion, is a crucial strategy for *Typha* blades to accommodate wind forces. Flow visualization demonstrates that the twisting morphology of blades provides advantages over the flat one in the context of two integrated functions: improving wind resistance and mitigating vortex-induced vibration. The unusual dynamic responses and superior mechanical properties of *Typha* blades are closely related to their biological/ecosystem functions and macro/micro structures. This work decodes the physical mechanisms of chirality-dependent flutter in *Typha* blades and holds potential applications in vortex-induced vibration suppression and the design of, e.g., bioinspired flight vehicles.

Historically, aquatic vegetation was viewed as no more than a source of hydraulic resistance that exacerbates flooding^1^ and impeded conveyance^2^. As a result, vegetation was often removed from river channels, streams, and canals^3^. However, it is now widely recognized that aquatic plants, albeit their side effects on water conservancy, can provide important ecosystem functions, e.g., improving water quality^4^, reducing erosion^5^, stabilizing river channels^6^, diminishing sediment suspension, and promoting particle retention within the canopy^7^,^8^. By resisting flow and altering local flow condition, vegetation creates habitat regions of low-flow for fish and aquatic invertebrates^9^,^10^.

The drag generated by aquatic macrophytes is important for ecosystem management. However, excessive drag may cause the rupture^2^ and dislodgment^11^ of plant stems. The survival of vegetation strongly depends on its strategies to accommodate external loads. Flow-adaptive reconfigurations, e.g., streamlining^12^, flapping^13^, bending and twisting^14^, are essential mechanisms for flexible plants to reduce their drag. The reconfiguration can be decomposed into a mean state and a fluctuation, which are referred to as the static reconfiguration (e.g., time-averaged streamlining and frontal surface reduction) and dynamic reconfiguration (plant flutter), respectively^15^.

The reconfiguration of a plant depends on the drag force and the restoring force due to its body stiffness^16^. Some aquatic plants can flexibly adjust their shapes to react to flow loads. Relative to the flexible species, such stiff, upright-standing plants as *Typha* rely less on the streamlining^17^,^18^ and thus could produce greater hydraulic resistance for ecosystem services. *Typha*, also named cattail, is an emergent aquatic macrophyte that can be found in almost any wet habitat around the world. *Typha* blades are featured by a distinct twisting chiral morphology. Their emergent lengths can reach as high as several meters. The slender blades must withstand the equal and opposite drag force exerted by wind. Thus an interesting question arises: What tactics do the *Typha* plants adopt to balance their biological functions (e.g., hydraulic resistance) and mechanical properties (e.g., lodging resistance)?

In this paper, we investigate the chirality-dependent flutter of twisting *Typha* blades in wind. Flapping is widely observed in natural biological materials. Flapping-wing aircraft, and bird and insect flight as well, have fascinated humans for many years^19^. A variety of deformation mechanisms, including modification and reversal of camber between upstroke and downstroke, twisting, area expansion and contraction, and transverse bending, might be involved in flapping flight^20^. Investigating the physical mechanisms of passive flapping or flutter can deepen our understanding on the structure–property–function relations of both animal wings and plant fronds. An attempt is here made to explore the aeroelastic properties of the pre-twisted *Typha* blades in wind. Their unusual fluttering behavior involving both wind-induced rotation and torsion is revealed through fluid dynamics simulations and wind tunnel experiments. Their interior structures are observed, and the relations between their chiral morphology and vortex-induced vibration (VIV) mitigation are investigated. The results demonstrate that the chirality-dependent mechanical properties of *Typha* blades could be closely related to their biological/ecosystem functions, macroscopic morphology, and microstructures.

## Results

The aeroelastic behavior of *Typha* blades was investigated through fluid dynamics simulations and wind tunnel experiments. The numerical and experimental results, combined with structural observation, allow us to uncover their structure–property–function relations of *Typha* blades and other aquatic plants.

**Figure 1:**
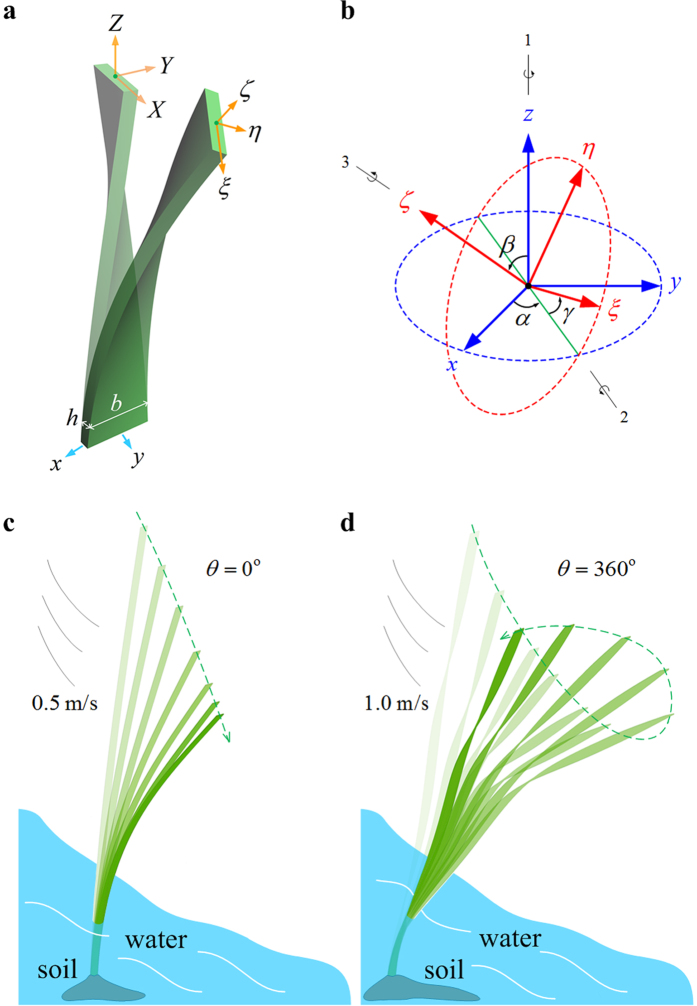
Model and results of fluid dynamics simulation. (**a**) A pre-twisted cantilever beam model of *Typha* blade, (**b**) the definition of Euler angles *α*, *β*, and *γ*, and the temporal evolution of deformed configurations of (**c**) a flat beam and (**d**) a 360°-twisted beam towards unidirectional airflow.

In our simulations, a *Typha* blade is modeled as a clamped-free beam exposed to an unidirectional incoming airflow. Refer to the fixed Cartesian coordinate system (*x*, *y*, *z*), as shown in Fig. 1a, where the coordinate origin *o* is located at the cross-sectional centroid at the bottom end of the beam; *x*, *y*, and *z* axes are along its width *b*, thickness *h*, and length *L* directions, respectively. In both the fluid dynamics simulations and wind tunnel experiments, the *x*, *y*, and *z* axes lay along the spanwise, streamwise, and wall-normal directions, respectively. Further, we establish the local coordinate systems (*X*, *Y*, *Z*) and (*ξ*, *η*, *ζ*), as shown in Fig. 1a .The former is attached to the blade in the reference configuration and does not move with the beam deformation, while the latter is defined in the current configuration and rotates with the beam together. The *Z* axis coincides with *z*, and the *ζ* axis is in the axial direction of the deformed beam. The deformation of the fluttering beam can be described by the transformation relations of the three coordinate systems (Fig. 1b).

### Wind-induced rotation

The pre-twisted *Typha* blades exhibit unusual fluttering behavior in wind. We first calculate the wind-induced rotation of a blade. A flat beam and a twisting cantilever-free beam towards unidirectional airflow are compared through fluid dynamics simulations in order to uncover whether or not the chiral morphology provides any functional advantages. Selected snapshots of deformed configurations of a flat beam and its twisted counterpart with *θ* = 360° are shown in Fig. 1c,d, respectively. In each plot, the transparency of configurations is decreased with time. The free-stream velocity *v* of the airflow is set as 0.5 m/s for the flat beam and 1.0 m/s for the twisted beam, such that their maximal deflections are similar in magnitude. The influence of water on the fluttering behavior of blades is neglected in the numerical simulations. Here and in the sequel, we refer to the cross-sectional centroid at the free end of the beam as its endpoint. The endpoint trajectories, represented by the green, dashed arrows, show the notably different dynamic responses of the beams.

**Figure 2:**
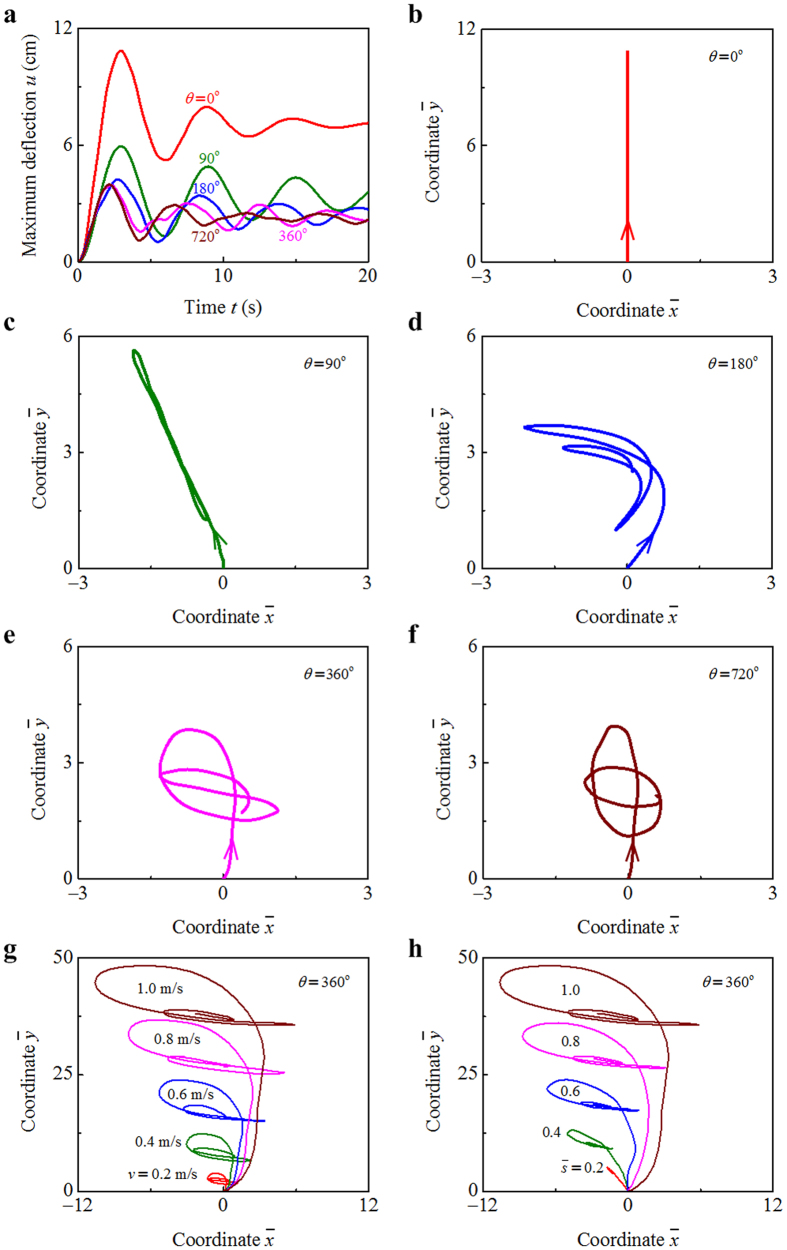
Rotational flutter of a chiral blade. (**a**) Temporal evolution of the maximum transient deflection *u* of the beams with different twist angles *θ*, and (**b**–**f**) their endpoint trajectories in the first ten seconds. (**g**) Endpoint trajectories of the 360°-twisted beam exposed to wind with different free-stream velocities, and (**h**) the trajectories of its cross-sectional centroids at different positions.

The temporal evolution of the maximum transient-state deflection *u* of the beam is plotted in Fig. 2a, where we take several representative twist angles *θ*. Both the deflection *u* and its vibration amplitude significantly decrease as *θ* increases from 0° to 360°. The deflection *u* is insensitive to the twist angle when the latter has a large value. For example, the 360° and 720°-twisted beams have the similar maximal deflections. Figure 2b–f give the endpoint trajectories of the beams in the first ten seconds, where the coordinates are normalized as 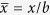 and 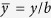. Each trajectory starts at the original point (0, 0), and the arrows indicate their evolutionary directions. The spanwise deflection keeps zero for a flat beam (*θ* = 0°), whereas it is nonzero for a pre-twisted beam (*θ* > 0°). The twist angle *θ* of a beam has a substantial influence on its endpoint trajectory. In the case of *θ* = 90°, the endpoint of the beam has an approximately linear trajectory (Fig. 2c). When the beam has a twist angle *θ* = 180°, its endpoint trajectory consists of several pieces of arcs (Fig. 2d). The endpoints of beams with *θ* = 360° and *θ* = 720° exhibit similar rotational fluttering behaviors (Fig. 2e,f).

Then we examine the dependence of the wind-induced rotation of the twisted beam on the flow velocity *v*. The beam has similar rotational behaviors as *v* increases from 0.2 m/s to 1.0 m/s (Fig. 2g). When the velocity *v* = 0.2 m/s, the maximum deflection *u* of the beam, ~0.04 m, is much smaller than the beam length *L* = 1 m (Fig. 2a). Rotation is the main deformation mechanism in the case of small deflection. On the other hand, the slender beam is pushed over into a significant streamlined posture when *v* = 1.0 m/s. This implies that the rotational flutter remains an important deformation mechanism for a twisted beam subjected to heavy wind. Let 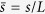 denote the normalized arc length coordinate of its central axis. The temporal evolutions of the centroids at different cross sections are further plotted in Fig. 2h, where we take *v* = 1.0 m/s and 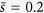, 0.4, 0.6, 0.8, and 1.0. It is seen that each cross section of the beam has a similar rotation.

### Wind-induced torsion

**Figure 3:**
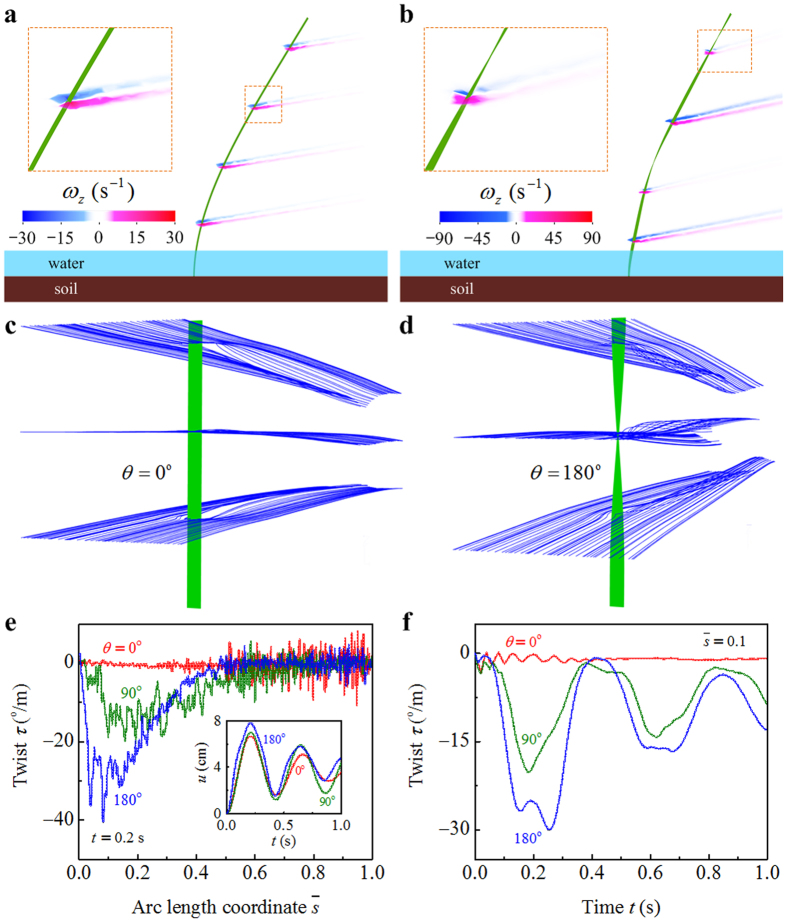
Torsional flutter of a chiral blade. Flow visualization of the wall-normal vorticity of fluid in the cases of (**a**) a flat beam and (**b**) a pre-twisted beam towards unidirectional airflow. Flow visualization of the path lines of fluid for (**c**) a flat beam and (**d**) a pre-twisted beam towards unidirectional airflow. Excess twist *τ* of three beams as functions of the (**e**) arc length coordinate and (**f**) time, where we take *θ* = 0°, 90°, and 180°.

We combine fluid dynamics simulations and wind tunnel experiments to explore the torsional flutter of *Typha* blades. The dependence of wind-induced torsion on the twisting chirality is first examined through fluid dynamics simulations. Figure 3a,b show a flat beam and a twisted beam in an airflow field, respectively. The free-stream velocity *v* is set as 0.5 m/s for the flat beam and 1.0 m/s for the twisted beam. The wall-normal vorticity of fluid is defined as


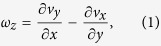


where *v*_*x*_ and *v*_*y*_ are the flow velocities in the *x* and *y* directions, respectively. It is found that the vorticity *ω*_*z*_ varies indistinctly with the *z* coordinate of the flat beam (Fig. 3a), while it changes distinctly for the twisted beam (Fig. 3b). For each height *z*, the contours of vorticity are divided into two groups in terms of their signs. The two groups of contours take similar shapes in the case of *θ* = 0° (Fig. 3a), but have distinctly different shapes when *θ* = 360° (Fig. 3b). Due to the variation in the angle of attack, the vorticity varies distinctly along the wall-normal direction. The path lines of fluid are visualized for two cantilever-free beams. The beams have the same length *L* = 200 mm, width *b* = 10 mm, thickness *h* = 1 mm, but different twist angles *θ* = 0° and 180°, and are subjected to the same incoming airflow with *v* = 0.1 m/s. For the flat beam, the flow field is approximately symmetric about the (*y*, *z*) plane (Fig. 3c). However, the symmetry is broken when the beam has a chiral morphology (Fig. 3d). The cross sections of the twisted beam are subjected to a torque induced by the asymmetrical flow field.

We compare three beams with the same length *L* = 1000 mm, width *b* = 20 mm, and thickness *h* = 0.5 mm, but with different twist angles *θ* = 0°, 90°, and 180°. For the three beams, the free-stream velocities of wind are ascertained as *v* = 2.8 m/s, 4 m/s, and 5 m/s, respectively, such that the three beams have the similar maximal deflections *u*. The excess twist of cross section, *τ*, is proportional to the torque that it faces. The excess twist *τ* is determined by combining numerical simulation and theoretical analysis, as plotted with respect to the arc length coordinate 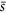 in Fig. 3e, where we take the time *t* = 0.2 s. The temporal evolution of the deflection *u* shows that the total deformations of the three beams peak at *t* ≈ 0.2 s (the inset of Fig. 3e). The 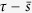 curves demonstrate that the wind-induced torsion is prominent in the pre-twisted beams (*θ* = 90° and *θ* = 180°). For the beams with twisting chirality, the torsional deformation of their basal part is larger than that of their forepart. By contrast, the torsion of the flat beam (*θ* = 0°) is indistinct. Only its forepart exhibit slight torsional deformation during flutter. The temporal evolution of the excess twist *τ* of the three beams is further plotted in Fig. 3f, where we take 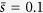. Due to the restoring force induced by torsional stiffness, the twist *τ* varies with *t* in an oscillatory manner. *τ* is smaller than 0 in the cases of *θ* = 90° and *θ* = 180°, indicating that the wind force tends to untwist the chiral beams. These results agree with the phenomena observed in the following experiments.

**Figure 4:**
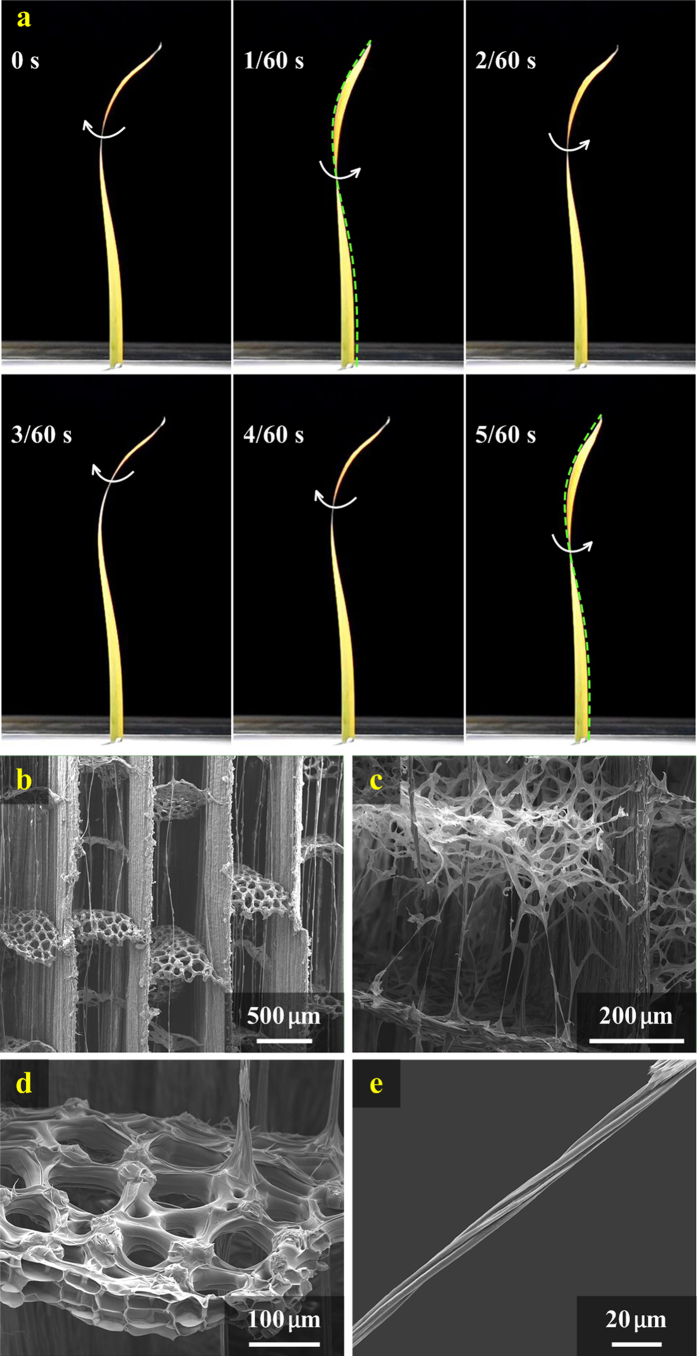
Experimental observations and measurements of *Typha* blade. (**a**) Selected snapshots of a *Typha* blade fluttering in wind. Microstructures of *Typha* blade observed using SEM, including (**b**) cuboid lacunae, (**c**) foam tissues, (**d**) fiber cables anchored in diaphragms, and (**e**) helical cellulose microfibrils of a fiber cable.

The wind-induced torsion of the pre-twisted blades was further investigated by wind tunnel experiment. The deformation of the blade was recorded as time-series graphs, as shown in Fig. 4a. The time interval between successive frames was 1/60 s. A white arrow was attached in each frame to represent the current torsional direction of the blade. A green, dashed line was used in the frames 1/60 s and 5/60 s to visualize the blade edge. It was found that the twisted blade had a notable torsional deformation in the flow field.

### Helical microstructure of *Typha* blades

To uncover the structure–property–function relations of *Typha* blades, we also observed their multiscale structures. The interior microstructures of a *Typha* blade are shown in Fig. 4b–e. A slender blade is divided into regular cuboid lacunae, which allow oxygen and carbon dioxide to be transported through the entire length of the blade (Fig. 4b). The thick partitions, referred to as mediastinum, separate the dorsal and ventral epidermis. The thin partitions, referred to as diaphragm, segment the long lacunae into shorter compartments. The foam tissues, filled in air chambers (lacunae) remote from the epidermis, connect the mediastinum and diaphragm (Fig. 4c). The fiber cables are anchored in diaphragm, which is composed of two or three layers of thin-walled stellate cells of aerenchyma tissue (Fig. 4d). Figure 4e clearly shows that the fiber cable is constructed by some microfibrils helically winded about its centerline.

### Stiffness and strength

**Figure 5:**
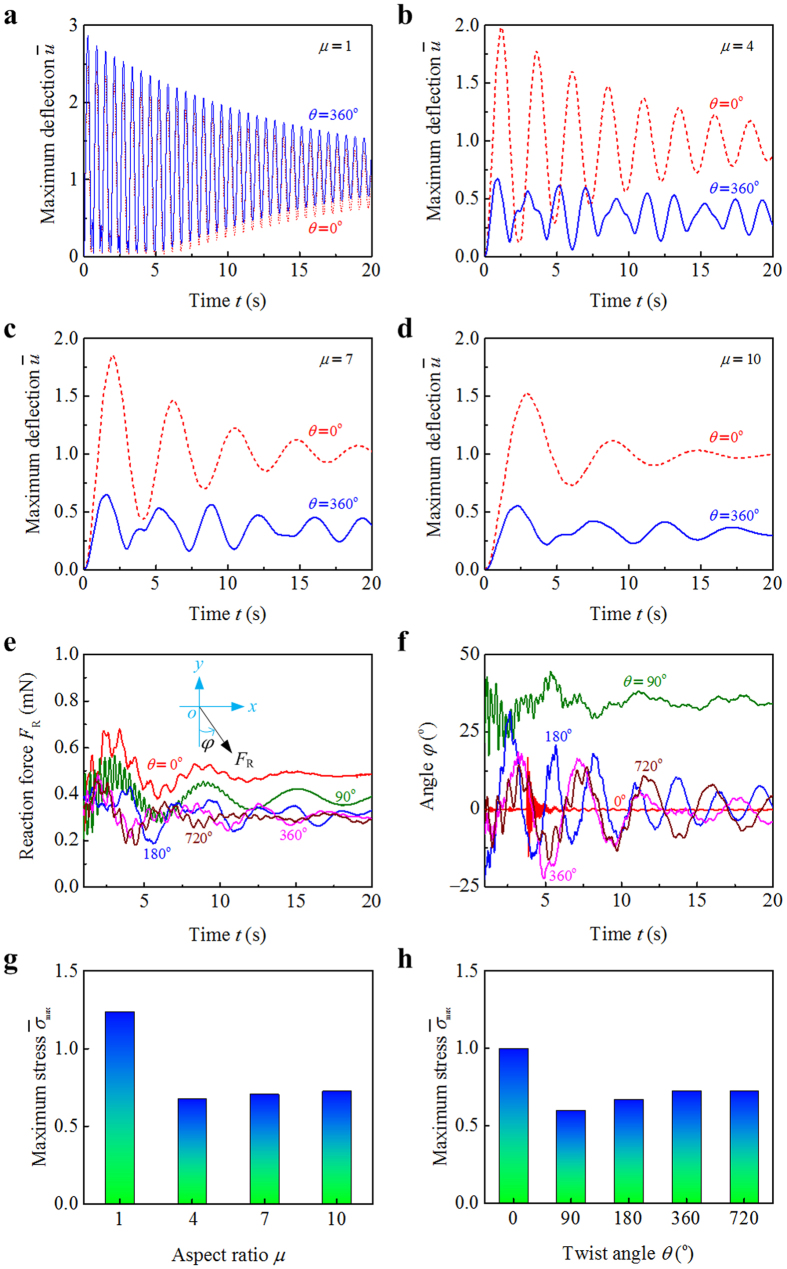
Effects of structural chirality on the mechanical properties of a chiral blade. Temporal evolutions of the maximum normalized deflection 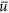 of the flat and pre-twisted beams, where we take the cross-sectional aspect ratio *μ* = 1 in (**a**), *μ* = 4 in (**b**), *μ* = 7 in (**c**), and *μ* = 10 in (**d**). Temporal evolutions of the (**e**) support reaction force *F*_R_ and (**f**) its direction *φ* for the beams with different twist angles. The normalized von Mises stress 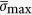 as functions of (**g**) the cross-sectional aspect ratio *μ* and (**h**) twist angle *θ*.

The effects of morphological chirality on the stiffness of beams with different structural parameters are further examined. Temporal evolution of the normalized deflection 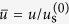 of the beams with *θ* = 0° and *θ* = 360° is plotted in Fig. 5a–d, where *u* is the maximum transient deflection and 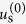 its steady-state value in the case of *θ* = 0°. Figure 5a shows that the chiral morphology interferes slightly with the deflection 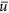 of a beam with square cross-section (*μ* = 1). Static analysis^21^ suggests that the stiffness of a pre-twisted beam does not vary with the twist angle when *μ* = 1. However, the drag coefficient of a beam strongly depends on its morphological chirality. The small difference in the 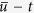 curves between the cases *θ* = 0° and *θ* = 360° is thus induced by the different aerodynamic effects. For a beam with larger aspect ratio *μ*, both the deflection 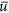 and its amplitude can be significantly decreased by twisting it into a chiral morphology (Fig. 5b–d). In addition, it is seen that as the aspect ratio *μ* increases, the frequency of deflection oscillation is significantly decreased, which could be related to the change in the structural natural frequency of vibration.

The temporal evolutions of the support reaction forces *F*_R_ acting on the beams are plotted in Fig. 5e. The flat beam (*θ* = 0°) has a greater windward area than the twisted beams, and correspondingly, it endures a distinctly larger force *F*_R_. The pre-twisted beams with *θ* = 90°, 180°, 360°, and 720° have the same windward area in the undeformed configuration. It is of interest to mention that the force *F*_R_ acting on the 90°-twisted beam is larger than those on the other three beams. This is mainly because the wind-induced torsion increases the windward area of the 90°-twisted beam. Let *φ* denote the angle of the reaction force measured from the negative direction of the *y* axis. The *φ* − *t* curves are plotted in Fig. 5f. The angle *φ* of the flat beam (*θ* = 0°) keeps nearly zero after *t* > 7 s, suggesting that the flow load it faces is always along the streamwise direction. The flow load acting on the 90°-twisted beam has a distinct inclination angle with the streamwise direction. In the cases of *θ* = 180°, 360°, and 720°, the *φ* − *t* curves varies in an oscillatory manner.

The strength of a beam could be evaluated according to the maximum stress it withstands. The maximum von Mises equivalent stress 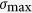 of a beam is normalized by that in the case of *θ* = 0°, which is designated as 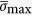. The variation in 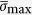 with respect to the cross-sectional aspect ratio *μ* and twist angle *θ* of a beam are plotted in Fig. 5g,h, respectively. When the beam has a narrow cross-section (e.g., *μ* = 4, 7, and 10), twisting it into a chiral morphology can substantially decrease the maximum stress (Fig. 5g). This effect will be saturated as the twist angle *θ* increases to a sufficiently large value, e.g., 360° (Fig. 5h). 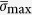 exceeds 1 in the case of *μ* = 1 and *θ* = 360°, which is attributed to the increasing drag force induced by the chiral morphology.

## Discussion

Plants with limited materials generally seek to maximize their surface area to gain sufficient sunlight for photosynthesis. By modelling a *Typha* blade as a rectangular beam (*L* ≫ *b*, *h*), its surface-area-to-volume ratio can be estimated by


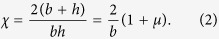


When the beam width *b* is fixed, the ratio *χ* increases linearly with the cross-sectional aspect ratio *μ*. It is thus reasonable for *Typha* blades to evolve a narrow cross section. However, the large aspect ratio *μ* of blades leads to a significant bending anisotropy. In order to lower their transverse bending anisotropy, *Typha* blades adopt chiral growth tactics.

The slender emergent *Typha* blades enable a variety of biological and ecosystem functions, e.g., conducting photosynthesis, adapting to water-level fluctuations, and providing hydraulic resistance. Besides optimizing interior structures, chiral growth is recognized as another robust strategy in *Typha* blades that helps them to achieve a sufficient height^22^. The stiff, upright-standing configuration of blades, however, leads to a notable drag force when they are exposed to wind. Excessive, drastic deformation of the wind-resilient blades might not only break their stems, but also perturb water and sediment fluxes in watersheds. By considering the fluid-structure interactions, we here demonstrate that the twisting chiral morphology is of importance for *Typha* blades to improve their survivability and realize their ecosystem functions. It is further found that *Typha* blades have evolved optimal inner structures to adapt the aerodynamic effects, e.g., wind-induced torsion.

### Relation between chiral morphology and biological functions

Due to the chiral morphology, the pre-twisted *Typha* blades have deflections in both the streamwise and spanwise directions in wind. For a clamped-free, twisted blade in wind, the support reaction force it withstands may distinctly deviate the upwind direction (Fig. 5f). The results of fluid dynamics simulation of the beams with and without twisting chirality reveal distinctly different dynamic responses (Fig. 1c,d). An untwisted beam exhibits a planar, reciprocating flutter in the streamwise direction, while the twisted beams show rotational fluttering behavior. Both the maximum transient-/steady-state deflections and variational amplitude of *Typha* blades can be significantly decreased by the rotational flutter, as shown in Fig. 2a. In comparison with the back-and-forth motion, the rotational flutter of blades can not only effectively decrease their maximum deformation, but also facilitate wave attenuation. Therefore, the morphological chirality of *Typha* blades provides distinct advantages to achieve their ecosystem functions, e.g., stabilizing water and diminishing sediment suspension.

### Relation between wind-induced torsion and interior microstructures

Wind-induced torsion is widely encountered in asymmetrical structures. For example, the pitch reversal of insect wings could be complemented without the aid of musculature^23^. The passive pitch torsion of wings is of vital importance to generate a downward propulsive force averaged over a full stroke cycle^24^. Understanding the physical mechanism of wind-induced torsion holds potential applications in advanced artificial flight vehicles, e.g., robotic insects^25^.

Here we attempt to reveal the relation between the wind-induced torsion and the interior microstructures of *Typha* blades. The shell-like epidermis is one of the main load-bearing element in a *Typha* blade. The Young’s modulus of epidermis varies from 0.5 GPa to 2 GPa, and the tensile strength from 12.7 MPa to 25.7 MPa^26^. The Young’s modulus and tensile strength of the fiber cables in *Typha* are 22.8 ± 7.4 GPa and 0.45 ± 0.17 GPa, respectively^27^. Both the stiffness and strength of the fiber cables are approximately one order of magnitude larger than those of the epidermis. The fiber cables traverse the lacunae of *Typha*, and their lengths are comparable to the blade length. Due to their superior mechanical properties and large length, the fiber cables have a considerable influence on the overall mechanical behaviors of *Typha* blades. In combination with the epidermis and mediastinum, which are strong in compression, the fiber cables, which are strong under tension, form a tensegrity structure^27^.

The fiber cables in *Typha* blades are comprised of cellulose microfibrils helically winded about their centerlines. Generally, helical fibers of plants play an important role in enabling biological functions and therefore, improving their survivability^28^,^29^. For example, multilayered and helically-wound bast fibers greatly promote the deformability and strength of bamboo^30^. The spiral fibers of trees can protect their stems against torsion-induced breakage in heavy wind when the externally applied torque is in the direction of the spirality^31^. The *Typha* blades, featured by a twisting chiral morphology, may undergo a distinct torsional deformation in wind (Fig. 3e,f), which is critical to their mechanical strength. Wind-induced torque (or excess twist) is always in the direction of the handedness of the fiber cables (Fig. 4). The helical assembly of cellulose microfibrils can effectively improve both the torsion resistance and strength of *Typha* blades.

### Relation between vortex-induced vibration mitigation and chiral morphology

When vortices are shed from a riser, the latter is subjected to a time-dependent vortex force. The vortex force may induce in-line and cross-flow vibrations, which are referred to as vortex-induced vibration (VIV)^32^. VIV mitigation of bluff structures is a challenging issue in riser and pipeline designs^33^. Suppression of vortex shedding and hence VIV is a major concern in the fields of buildings, bridges, chimneys, and subsea tubulars. Helical strake is a popular control for VIV suppression in a variety of industrial and offshore applications. By attaching three-start helical strakes to a riser, its vibration amplitude can be suppressed by as great as 98%^34^. Helical strakes not only destroy regular vortex shedding in the streamwise direction, but also prevent the shedding from becoming correlated in the wall-normal direction^35^.

*Typha* blades are featured by a chiral morphology at the macroscale. As wind past a pre-twisted blade, its helical edges, analogous to the spiral strakes attached to a riser, chop up the flow and create vortices at various places along the wall-normal direction. The vortices might be out of phase with each other. We performed flow visualization to uncover the relation between VIV and the chiral morphology of *Typha* blades. Two cantilever-free beams are compared. The smooth wakes behind the untwisted beam and the undulating wakes behind the pre-twisted beam are clearly illustrated in Fig. 3c,d, respectively. The fluid is channeled by the helical edges of the pre-twisted beam, rendering helical separation points and further a three-dimensional flow downstream. However, the separation occurs uniformly along the wall-normal direction of the flat beam. The turbulent wake formed in the back of the pre-twisted beam breaks the vortex coherence and thus mitigates the VIV. In the streamwise direction, the helical edges of the beam weaken the interaction between the two shear layers due to separation. Therefore, the VIV mitigation in *Typha* blades lies in two physical mechanisms. One is to adversely affect the shear layer to roll up, and the other is to disrupt the vortex formation and shedding in the wall-normal direction.

## Conclusion

In summary, we have investigated, through a combination of experimental measurements, fluid dynamics simulations, and theoretical analysis, the aeroelastic behavior of *Typha* blades. The wind-induced flutter of blades, including rotation and torsion, is found to be strongly dependent of their chiral morphology. In comparison with reciprocation motion, the rotational flutter of the blades not only protect their stems from lodging failure by decreasing the maximum deformation, but also ensures their ecosystem functions by facilitating wave attenuation. The fiber cables in *Typha* blades are found with prominent helical microstructures, rendering an enhanced resistance to accommodate wind-induced torsion. Flow visualization demonstrates that the twisting chiral morphology of a *Typha* blade can effectively mitigate vortex-induced vibration. This work deepens our understanding of the chirality-dependent fluttering behavior of *Typha* blades in wind, and holds potential applications in vortex-induced vibration suppression and the design of bioinspired flapping vehicles and aerocrafts.

## Methods

### Fluid dynamics simulations

Fluid dynamics simulations were performed by using the commercial software ANSYS (Version 15.0, ANSYS Inc., USA). The *Typha* blade was modeled as a cantilever-free beam exposed to an unidirectional incoming airflow. Assume that the beam has a solid, rectangular cross-section and an intrinsic twist angle from its base to apex. The two-way coupling method is used for the numerical analysis of fluid-structure interaction. In each iteration, the wind pressure acting on the beam is transferred to the structure solver, and then the deformation of the beam is passed back to the fluid solver. At the fluid-structure interface, the information is shared between the two solvers. The parameter settings of the fluid dynamics simulation are listed in Table 1. In the default cases, we take the beam length *L* = 1000 mm, width *b* = 10 mm, thickness *h* = 1 mm (corresponding to the cross-sectional aspect ratio *μ* = *b*/*h* = 10), twist angle *θ* = 360°, and free-stream velocity *ν* = 0.2 m/s.

### Theoretical analysis

On the basis of the fluid dynamics simulations, a theoretical model is here established to reveal the torsional flutter of *Typha* blades in wind. The beam has a twist angle 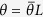 in the absence of external force, where 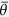 denotes the twist angle per unit length along the longitudinal direction. Assume that the beam is inextensible. The arc length coordinate of its central axis is denoted as *s*. Let {**d**_1_,**d**_2_,**d**_3_} represent the orthonormal unit basis vectors of the system (*ξ*, *η*, *ζ*). The derivative of **d**_i_ (*i* = 1, 2, 3) with respect to *s* is defined as


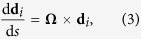


where **Ω** = Ω_*i*_**d**_*i*_ is the curvature-twisting vector of the deformed beam. Ω_1_ and Ω_2_ are the curvatures of the projections of the deformed central axis on the planes (**d**_2_, **d**_3_) and (**d**_1_, **d**_3_), respectively. Ω_3_ is the local twist of the beam about its axis. Here and in the sequel, Einstein summation convention is used and all dummy indexes vary from 1 to 3. From Eq. (3), the components Ω_*i*_ of curvature-twisting vector can be expressed as


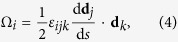


where *ε*_*ijk*_ is the permutation symbol. To determine the deformation Ω_*i*_ of the beam, we first need to solve the rotations of its cross sections.

Introduce the Euler angles *α*, *β*, and *γ* to describe the rotation of the frame (*ξ*, *η*, *ζ*) with respect to (*x*, *y*, *z*), as shown in Fig. 1b. The rotation matrix takes the form as^36^


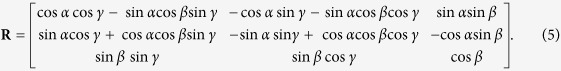


Denote the orthonormal unit basis vectors of the system (*x*, *y*, *z*) as {**e**_1_, **e**_2_, **e**_3_}. Then one has


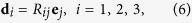


where *R*_*ij*_ are the elements of the rotation matrix **R**. Substituting Eqs (5) and (6) into (4) leads to


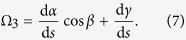


From Eq. (5), the Euler angles can be expressed as


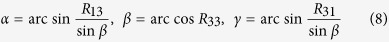


where the singular case sin *β*  = 0 is ruled out.

Notice that the beam has an intrinsic twisting 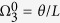, the excess twist *τ* induced by deformation is


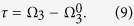


Assume that the material is linear elastic and isotropic. The torque that the beam faces is calculated as


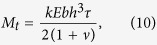


where *E* is the Young’s modulus, *ν* the Poisson’s ratio, and *k* a dimensionless function of the cross-sectional aspect ratio *μ*^37^. Thus, once the deformed configuration of the beam is determined from the fluid dynamics simulations, we can calculate its torsional deformation using the above equations.

### Wind tunnel experiments

Wind tunnel experiments were carried out to investigate the aerodynamic behaviors of the twisted blades. Fresh, mature blades of *Typha orientalis* C. Presl were collected in Beijing, China. They were used in both the wind tunnel experiments and the structure observation. The low-speed wind tunnel has a square test section of 0.5 m × 0.5 m and a length of 2.0 m. The wind velocity *v* was set as 3 m/s and the turbulence intensity was less than 0.5% in the free steam. The specimen was made from the forepart of a *Typha* blade. It has a length of 0.25 m and a twist angle of about 180° in the absence of external loads. The width and thickness of its bottom cross-section are approximately 1.0 cm and 0.3 cm, respectively. The specimen was vertically mounted with its base clamped at the bottom of the working section. At the clamped end, the width face of the specimen was faced to the incoming flow. A digital camera (D750, Nikon Co., Japan) was located downstream and far from the working section to capture the deformation of the specimen.

### Structure observation

The interior microstructures of *Typha* blade were observed by using a scanning electron microscope (SEM, Quanta FEG 450, FEI, USA). The blade samples were successively cleaned with ionized water, frozen at −80 °C, dried in a freeze-drier device (FD-1A-50, Beijing Boyikang Medical Equipment Co., P.R. China), and gold sputtered before observation. In order to observe their interior structures, we removed the epidermis of the samples.

## Additional Information

**How to cite this article**: Zhao, Z.-L. *et al*. Chirality-dependent flutter of *Typha* blades in wind. *Sci. Rep*. **6**, 28907; doi: 10.1038/srep28907 (2016).

## Figures and Tables

**Table 1:** Parameters used in the fluid dynamics simulations.

Beam		Airflow	
Mass density (kg·m^–3^)	1000	Mass density (kg·m^–3^)	1.23
Length *L* (mm)	1000	Free-stream velocity *v* (m·s^–1^)	0.2, 0.4, 0.6, 0.8, 1
Width *b* (mm)	10	Dynamic viscosity (kg·m^–1^·s^–1^)	1.79 × 10^–5^
Thickness *h* (mm)	1, 1.43, 2.5, 10	Domain size *x* × *y* × *z* (mm × mm × mm)	400 × 900 × 2000
Twist angle *θ* (^o^)	0, 90, 180, 360, 720	Turbulent intensity	3%
Young’s modulus *E* (GPa)	1	Computational time step (s)	0.01
Poisson’s ratio *ν*	0.3	Turbulence modeling	*k*–*ε* model
Element type	tetrahedron, 10 nodes	Element type	tetrahedron
Element number	~5.0 × 10^4^	Element number	~1.0 × 10^7^

## References

[b1] Kouwen N. & Unny T. E. Flexible roughness in open channels. J. Hydraul. Division 99, 713–728 (1973).

[b2] Duan J. G., French R. H. & Miller J. The lodging velocity for emergent aquatic plants in open channels. J. Am. Water Resour. Assoc. 38, 255–263 (2002).

[b3] Luhar M. & Nepf H. M. From the blade scale to the reach scale: a characterization of aquatic vegetative drag. Adv. Water Resour. 51, 305–316 (2013).

[b4] Costanza R. . The value of the world’s ecosystem services and natural capital. Nature 387, 253–260 (1997).

[b5] Lightbody A. F. & Nepf H. M. Prediction of velocity profiles and longitudinal dispersion in salt marsh vegetation. Limnol. Oceanogr. 51, 218–228 (2006).

[b6] Tal M. & Paola C. Dynamic single-thread channels maintained by the interaction of flow and vegetation. Geology 35, 347–350 (2007).

[b7] Neumeier U. & Ciavola P. Flow resistance and associated sedimentary processes in a *Spartina maritima* salt-marsh. J. Coast. Res. 20, 435–447 (2004).

[b8] Luhar M. & Nepf H. M. Flow-induced reconfiguration of buoyant and flexible aquatic vegetation. Limnol. Oceanogr. 56, 2003–2017 (2011).

[b9] Kemp J. L., Harper D. M. & Crosa G. A. The habitat-scale ecohydraulics of rivers. Ecol. Eng. 16, 17–29 (2000).

[b10] Bouma T. J. . Trade-offs related to ecosystem engineering: a case study on stiffness of emerging macrophytes. Ecology 86, 2187–2199 (2005).

[b11] Schutten J., Dainty J. & Davy A. Root anchorage and its significance for submerged plants in shallow lakes. J. Ecol. 93, 556–571 (2005).

[b12] Harder D. L., Speck O., Hurd C. L. & Speck T. Reconfiguration as a prerequisite for survival in highly unstable flow-dominated habitats. J. Plant Growth Regul. 23, 98–107 (2004).

[b13] Shelley M. J. & Zhang J. Flapping and bending bodies interacting with fluid flows. Annu. Rev. Fluid Mech. 43, 449–465 (2011).

[b14] Vogel S. Living in a physical world XI. To twist or bend when stressed. J. Biosci. 32, 643–656 (2007). 17762137 10.1007/s12038-007-0064-6

[b15] Siniscalchi F. & Nikora V. Dynamic reconfiguration of aquatic plants and its interrelations with upstream turbulence and drag forces. J. Hydraul. Res. 51, 46–55 (2013).

[b16] Alben S., Shelley M. & Zhang J. Drag reduction through self-similar bending of a flexible body. Nature 420, 479–481 (2002). 12466836 10.1038/nature01232

[b17] Koehl M. A. R. How do benthic organisms withstand moving water? Am. Zool. 24, 57–70 (1984).

[b18] Vogel S. Life in Moving Fluids: The Physical Biology of Flow. (Princeton University Press, 1996).

[b19] Alexander R. M. The U, J and L of bird flight. Nature 390, 13–13 (1997).

[b20] Shyy W., Berg M. & Ljungqvist D. Flapping and flexible wings for biological and micro air vehicles. Prog. Aeosp. Sci. 35, 455–505 (1999).

[b21] Zhao Z. L., Zhao H. P., Chang Z. & Feng X. Q. Analysis of bending and buckling of pre-twisted beams: A bioinspired study. Acta Mech. Sin. 30, 507–515 (2014).

[b22] Zhao Z. L. . Biomechanical tactics of chiral growth in emergent aquatic macrophytes. Sci. Rep. 5, 12610 (2015). 26219724 10.1038/srep12610 PMC4518234

[b23] Bergou A. J., Xu S. & Wang Z. Passive wing pitch reversal in insect flight. J. Fluid Mech. 591, 321–337 (2007).

[b24] Ma K. Y., Chirarattananon P., Fuller S. B. & Wood R. J. Controlled flight of a biologically inspired, insect-scale robot. Science 340, 603–607 (2013). 23641114 10.1126/science.1231806

[b25] Whitney J. P. & Wood R. J. Aeromechanics of passive rotation in flapping flight. J. Fluid Mech. 660, 197–220 (2010).

[b26] Zhao Z. L. . Synergistic effects of chiral morphology and reconfiguration in cattail leaves. J. Bionic Eng. 12, 634–642 (2015).

[b27] Witztum A. & Wayne R. Fibre cables in the lacunae of *Typha* leaves contribute to a tensegrity structure. Ann. Bot. 113, 789–797 (2014). 24532647 10.1093/aob/mcu002 PMC3962244

[b28] Zhao Z. L., Zhao H. P., Wang J. S., Zhang Z. & Feng X. Q. Mechanical properties of carbon nanotube ropes with hierarchical helical structures. J. Mech. Phys. Solids 71, 64–83 (2014).

[b29] Zhao Z. L., Li B. & Feng X. Q. Handedness-dependent hyperelasticity of biological soft fibers with multilayered helical structures. Int. J. Non-Linear Mech. 81, 19–29 (2016).

[b30] Parameswaran N. & Liese W. On the fine structure of bamboo fibres. Wood Sci. Technol. 10, 231–246 (1976).

[b31] Skatter S. & Kučera B. Spiral grain–an adaptation of trees to withstand stem breakage caused by wind-induced torsion. Holz als Roh-und Werkstoff 55, 207–213 (1997).

[b32] Blevins R. D. Flow-induced Vibration. 2nd ed. (Krieger Publishing, 2001).

[b33] Korkischko I. & Meneghini J. R. Experimental investigation of flow-induced vibration on isolated and tandem circular cylinders fitted with strakes. J. Fluids Struct. 26, 611–625 (2010).

[b34] Zhou T., Razali S. F. M., Hao Z. & Cheng L. On the study of vortex-induced vibration of a cylinder with helical strakes. J. Fluids Struct. 27, 903–917 (2011).

[b35] Gao Y., Fu S. X., Ma L. X. & Chen Y. F. Experimental investigation of the response performance of VIV on a flexible riser with helical strakes. Ships Offshore Struct. 11, 113–128 (2014).

[b36] Liu Y. Z. Nonlinear Mechanics of Thin Elastic Rod – Theoretical Basis of Mechanical Models of DNA. (Tsinghua University Press, 2006).

[b37] Timoshenko S. P. & Goodier J. N. Theory of Elasticity. (McGraw-Hill, 1969).

